# Special Issue “Molecular Progression in Genome-Related Diseases”

**DOI:** 10.3390/ijms27031184

**Published:** 2026-01-24

**Authors:** Salvatore Saccone, Francesco Calì

**Affiliations:** 1 Department of Biological, Geological and Environmental Sciences, University of Catania, 95124 Catania, Italy; 2 Oasi Research Institute-IRCCS, 94018 Troina, Italy; cali@oasi.en.it; 3 Department of Medicine and Surgery, Kore University of Enna, 94100 Enna, Italy

## 1. Background and Scope of the Special Issue

The landscape of molecular research into genome-related diseases has evolved rapidly in recent years, driven by advances in next-generation sequencing (NGS), multi-omics integration, and computational approaches. Genetic heterogeneity, epigenetic regulation, and gene–environment interactions are now recognized as central determinants of disease onset, progression, and interindividual variability in clinical outcomes [1,2,3,4,5,6,7,8]. Despite these advances, significant gaps remain in elucidating disease mechanisms and, critically, in translating genomic insights into reliable diagnostic tools, prognostic markers, and effective therapeutic strategies [9,10,11,12]. In parallel, ongoing efforts to refine genomic references are reshaping our ability to capture genetic diversity and molecular complexity. These include the development of the human pangenome and the emergence of spatiotemporal omics frameworks, which enable the characterization of dynamic molecular states across populations and tissues [13,14,15].

With this Special Issue, *Molecular Progression in Genome-Related Diseases*, we aimed to provide an integrated overview of current research efforts that bridge molecular discovery and clinical application across a wide spectrum of genome-driven disorders. The collected articles reflect both original research and focused reviews that address key biological, technological, and translational dimensions of genome-related diseases. The contributions collectively reflect how genomic technologies are reshaping our understanding of disease biology while also highlighting the challenges that must be addressed to move toward precision medicine.

## 2. Multi-Omics Approaches and Molecular Architecture of Disease

Some contributions in this Special Issue emphasize the power of multi-omics analyses in dissecting the molecular architecture of complex diseases. Several original articles focusing on hematological malignancies demonstrate how integrated transcriptomic and genomic profiling can uncover novel regulatory networks, mutational patterns, and disease-specific signatures, offering refined tools for patient stratification and prognostic assessment. These studies provide concrete examples of how multi-layered molecular data can improve disease characterization at both biological and clinical levels. From a broader methodological perspective, these advances align with the development of multi-omics integration frameworks that leverage machine learning and deep generative models to enhance interpretability and predictive power in disease classification [16]. Complementary reviews and original articles further illustrate how advances in NGS workflows and data interpretation are enabling more accurate molecular diagnoses, particularly in clinically heterogeneous conditions. Together, these works highlight the importance of harmonizing technological innovation with robust analytical frameworks to ensure clinical reliability. Moreover, the evolution of third-generation and long-read sequencing technologies continues to expand the analytical repertoire for resolving complex genomic structures and variants relevant to disease biology [17]. Recent large-scale multi-omics efforts further demonstrate how integrated molecular profiling can redefine disease subtypes. By linking genomic, transcriptomic, and metabolic layers with clinical phenotypes, these approaches enhance disease stratification and prognostic accuracy, with clear translational implications [18].

## 3. Gene–Environment Interactions and Exposome-Driven Modulation

A second major theme emerging from this Special Issue concerns the interplay between genomic susceptibility and environmental exposure. Contributions addressing epigenetic regulation and exposome-related effects highlight how external factors can shape gene expression programs and influence disease risk from early development through adulthood. Emerging work in translational exposomics underscores the importance of incorporating life-course exposures to better understand how environmental factors and genomic background jointly determine disease trajectories. At the same time, these studies emphasize persistent methodological challenges, particularly in data integration, standardization, and harmonization across heterogeneous datasets [19]. Whole-genome sequencing studies using model organisms further contribute mechanistic insights into environmentally induced mutational signatures relevant to human cancer development. These findings reinforce the concept that genome-related diseases cannot be fully understood without considering environmental context and regulatory plasticity. Importantly, contemporary toxicogenomics perspectives underscore how environmental factors influence fundamental processes such as cytochrome P450-mediated metabolism and genomic instability, bridging chemical exposures and disease susceptibility [20].

## 4. Neurodevelopmental and Neurodegenerative Disorders

Neurogenomic research represents another central pillar of this Special Issue. Contributions focusing on neurodevelopmental and epileptic encephalopathies underscore the clinical impact of whole-exome sequencing as a first-line diagnostic tool, enabling precise genotype–phenotype correlations and the identification of novel disease entities, including newly described SNAREopathies. In parallel, reviews addressing neurodegenerative disorders such as Alzheimer’s disease explore the contribution of both common and rare genetic variants, as well as epigenetic mechanisms, to disease progression and therapeutic vulnerability. These insights resonate with current syntheses of multi-omics and genomic strategies in precision medicine, emphasizing the translation of molecular signatures into neurologic care pathways [21]. Collectively, these studies exemplify the translational potential of neurogenomics, where early and accurate molecular diagnosis can substantially influence clinical management.

## 5. Translational Genomics and Emerging Therapeutic Strategies

Beyond diagnosis, several contributions highlight the growing translational dimension of genomic medicine. Articles addressing inherited disorders and cancer-related pathways illustrate how genomic insights are informing emerging therapeutic strategies, including allele-specific targeting, early-stage gene therapies, and personalized intervention models. Parallel developments in pharmacogenomics underscore the value of identifying genetic determinants of drug response to improve safety and efficacy, particularly in oncology and precision therapeutics [16,22]. Research on somatic mosaicism and genotype-driven treatment decisions further emphasizes the need for precise molecular characterization to guide therapeutic development. Collectively, these studies reflect a shift from descriptive genomics toward clinically actionable molecular medicine. At the same time, significant translational hurdles remain, including barriers to broad clinical implementation of pharmacogenomic testing and the integration of genomic biomarkers into real-world treatment pathways [23].

## 6. Outlook and Future Directions

The articles gathered in this Special Issue underscore the necessity of integrative genomic research to fully capture the complexity of genome-related diseases. Looking ahead, several key directions emerge. First, the integration of single-cell technologies with bulk genomic data promises to resolve cellular heterogeneity and dynamic regulatory processes underlying disease progression. Second, continued efforts toward standardization of variant interpretation and data sharing will be essential to translate molecular findings into consistent clinical practice. Finally, as gene-targeted and genome-editing therapies continue to advance, ethical, regulatory, and long-term safety considerations must be addressed alongside technological development. Advances in AI-driven omics integration and large-scale data harmonization are likely to further accelerate discovery and translational pipelines. However, these developments must be accompanied by equitable frameworks for access, implementation, and clinical adoption [16]. Recent computational multi-omics studies illustrate how integrative modeling of genomic, epigenomic, and transcriptomic data can uncover dynamic regulatory programs underlying complex biological states and disease progression, highlighting the growing role of systems-level approaches in genomic medicine [24]. In this context, coordinated international efforts, shared infrastructures, and interdisciplinary collaboration will be critical to ensure that genomic innovation translates into sustainable clinical benefit.

We are honored to present this collection of contributions, which collectively reflect the diversity, depth, and translational relevance of contemporary research in genome-related diseases. We sincerely thank all the authors, reviewers, and editorial staff for their invaluable contributions and commitment. We hope that this Special Issue will stimulate further research, foster interdisciplinary collaboration, and support the continued evolution of genomic medicine toward more precise and effective healthcare solutions.
